# Sources of plutonium in the atmosphere and stratosphere-troposphere mixing

**DOI:** 10.1038/srep15707

**Published:** 2015-10-28

**Authors:** Katsumi Hirose, Pavel P. Povinec

**Affiliations:** 1 Department of Materials and Life Sciences, Sophia University, Tokyo, Japan; 2 Department of Nuclear Physics and Biophysics, Comenius University, Bratislava, Slovakia

## Abstract

Plutonium isotopes have primarily been injected to the stratosphere by the atmospheric nuclear weapon tests and the burn-up of the SNAP-9A satellite. Here we show by using published data that the stratospheric plutonium exponentially decreased with apparent residence time of 1.5 ± 0.5 years, and that the temporal variations of plutonium in surface air followed the stratospheric trends until the early 1980s. In the 2000s, plutonium and its isotope ratios in the atmosphere varied dynamically, and sporadic high concentrations of ^239,240^Pu reported for the lower stratospheric and upper tropospheric aerosols may be due to environmental events such as the global dust outbreaks and biomass burning.

Atmospheric behavior of anthropogenic radionuclides, which originated from atmospheric nuclear weapons tests, satellite accidents and nuclear reactor accidents has been frequently studied both in terrestrial and marine environment during the past 50 years^1^,^2^. Large quantities of radionuclides were released into the atmosphere during atmospheric tests of nuclear weapons conducted by USA and former Soviet Union, mainly during the 1950s and early 1960s. During the large-scale nuclear weapons tests of hydrogen bombs, most of the radioactive debris reached the stratosphere, which became then the main reservoir of bomb-produced radionuclides. Exchange processes between the stratosphere and the troposphere, especially during the late spring when rising hot air provokes the descent of cold air masses from the lower stratosphere, enhanced concentrations of radionuclides in the troposphere (global fallout), producing regularly observed spring maxima in radionuclide concentrations in the mid-latitude regions.

After the moratorium on the atmospheric nuclear weapons tests signed in 1963, the new supply of bomb-produced radionuclides to the stratosphere was limited because of only minor contributions from atmospheric nuclear weapons tests conducted by France and China up to 1980. Therefore maxima in concentrations of bomb-produced radionuclides (e.g. ^14^C with a half life of 5,730 years; ^90^Sr with a half-life of 28 years; ^137^Cs with a half life of 30 years; ^238^Pu with a half life of 87.74 years; ^239^Pu with a half life of 24,100 years; ^240^Pu with a half life of 6,560 years and ^241^Pu with a half life of 14.4 years) in the lower troposphere were observed in 1963 (ref. 1, 2, 3). The typical spring maxima in the ground-level air caused by the stratosphere-troposphere inputs were observed till the 1990s. Later, the observed radionuclide variations have been mostly due to their resuspension from soil^2^,^4^,^5^,^6^,^7^,^8^. It has been believed therefore that there have not been significant amounts of these radionuclides left in the stratosphere because most of the radionuclides derived from the atmospheric nuclear testing conducted from 1945 to 1980 had already been transported to the lower troposphere and deposited on land and ocean surface^2^,^9^.

Stratospheric residence time of radioactive aerosols is an important concept characterizing stratospheric behavior of particles. In the 1960s, longer mean residence times of the order of 1–4 years were specified to bomb-derived radioactive aerosols in the stratosphere^10^,^11^,^12^. After 1970, a shorter stratospheric residence time (1–2 y) was determined from temporal changes of stratospheric distributions of bomb-derived radionuclides^13^, as well as from long-term continuous monitoring of their concentrations in ground-level air^3^,^14^. Models related to stratospheric transport were developed, and the stratospheric residence time of gaseous chemical components such as CO_2_ and SF_6_, were calculated. The obtained residence time of gaseous chemicals in the mid-latitude stratosphere was in the range of 1.1 to 2.1 y (ref. 15).

Recently Corcho Alvarado *et al.*^16^ reported interesting results of investigations of plutonium isotopes and ^137^Cs in stratospheric and tropospheric aerosols, which included new data observed during the period of 2007 to 2011. They found higher ^239,240^Pu and ^137^Cs concentrations, and higher ^238^Pu/^239,240^Pu activity ratios in the lower stratosphere and lower troposphere than expected. The observed levels of ^239,240^Pu in stratospheric aerosols were from two to four orders of magnitude higher than that in the ground-level air. They also suggested that the stratospheric mean residence time of plutonium and ^137^Cs should be 2.5–5 y, arguing that radionuclides attached to fine aerosol particles (<0.02 μm in diameter) could have a longer stay in the stratosphere, and therefore radionuclides injected there mainly during the early 1960s have still been present during the 2000s in the stratosphere. However, studying long-term variations (1964–2010) of plutonium isotopes in the stratosphere and surface air of the Northern Hemisphere we have found that the dominant processes affecting plutonium concentrations in the upper troposphere should be global dust events and biomass burning, and that its apparent residence time in the atmosphere did not change from 1.5 ± 0.5 years.

## Results

### Sources of plutonium in the atmosphere

The ^238^Pu, ^239^Pu, ^240^Pu and ^241^Pu represent the major plutonium isotopes released to the atmosphere during the atmospheric nuclear test^1^. Their isotope ratios (^238^Pu/^239,240^Pu, ^240^Pu/^239^Pu, ^241^Pu/^239,240^Pu) are powerful tools to elucidate sources of plutonium in the environment. For nuclear bomb-derived plutonium (global fallout), the ^238^Pu/^239,240^Pu activity ratio has been 0.03, the ^241^Pu/^239,240^Pu activity ratio in 1963 was 13–15 (ref. 17,18), and the ^240^Pu/^239^Pu atom ratio has been 0.18 (ref. 19, 20, 21). The plutonium isotope ratios derived from the nuclear weapons tests depend on explosion yields and the plutonium isotope composition of fissile materials. High ^241^Pu/^239,240^Pu activity ratios (27–30; ref. 20,22), high ^240^Pu/^239^Pu atom ratios (0.3; ref. 20,23, 24, 25, 26), and lower ^238^Pu/^239,240^Pu activity ratios (<0.01; ref. 20) were observed after high-yield thermonuclear tests carried out by US in the 1950s, whereas lower ^240^Pu/^239^Pu atom ratios (0.036–0.063) appeared in low yield tests carried out by US (Nevada test site)^27^,^28^ and former USSR (Semipalatinsk test site)^29^.

Nuclear power plant accidents such as Chernobyl and Fukushima were also sources of plutonium in the environment, although they were much lower scale events. Plutonium isotopes released from these accidents were characterized by higher ^238^Pu/^239,240^Pu, ^241^Pu/^239,240^Pu activity ratios and higher ^240^Pu/^239^Pu atom ratios than those ratios derived from nuclear tests^6^. The ^238^Pu/^239,240^Pu, ^241^Pu/^239,240^Pu activity ratios and ^240^Pu/^239^Pu atom ratio for the Chernobyl accident were 0.5, 85, and 0.41, respectively^17^,^30^,^31^, while for the Fukushima accident these ratios were 1.2, 108, and 0.30–33, respectively^32^,^33^. The plutonium isotopic signature may help therefore to better understand sources of anthropogenic radionuclides, and their behavior in the upper and lower atmosphere.

For better understanding of plutonium levels in the stratosphere it is therefore important to elucidate sources of the bomb-derived plutonium and other radionuclides observed in stratospheric aerosols in the 2000s. Possible radionuclide sources are the large-scale nuclear tests carried out in 1961–62, the Chinese nuclear tests (especially those conducted in 1976 and 1980), resuspension of plutonium from deserts, and biomass burning. To achieve this aim, we collected data of plutonium isotope concentrations in stratospheric air and in surface air in the Northern Hemisphere. Unfortunately, a continuous data set of both stratospheric and ground-level plutonium levels during the period of 1960–2010 has not been possible to construct. We describe here long-term variations (1964–2010) of plutonium isotopes in the stratosphere and surface air of the Northern Hemisphere using available data.

### Temporal variations of ^239,240^Pu in the stratosphere and surface air

**Figure 1:**
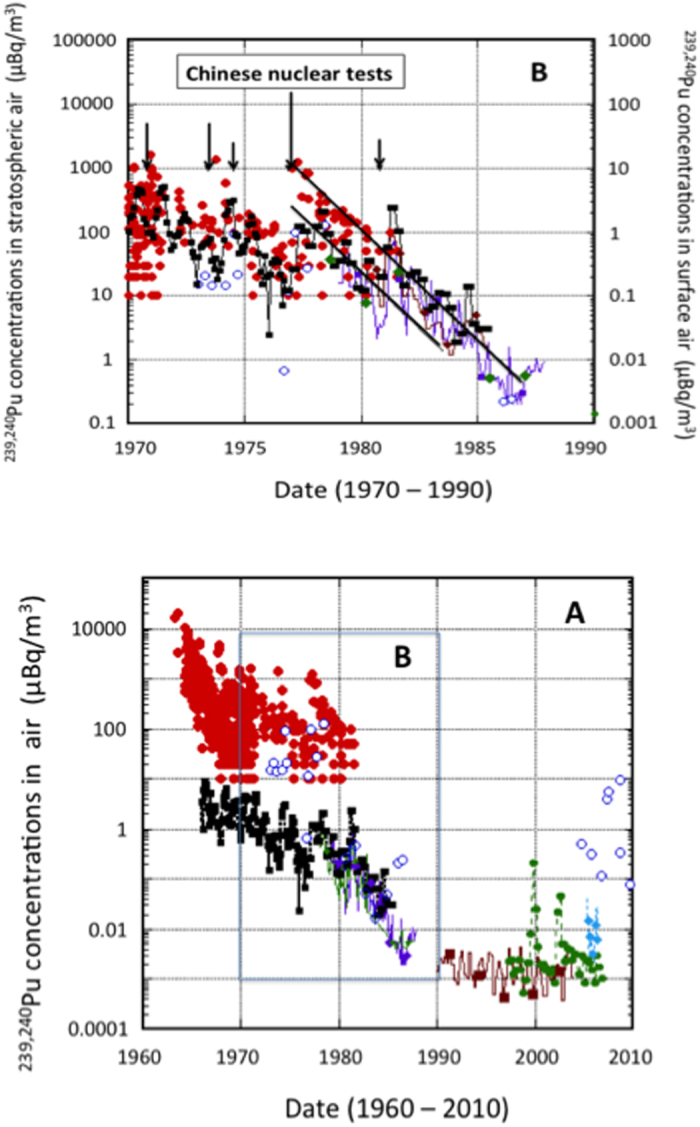
Temporal variations of ^239,240^Pu concentrations in stratospheric and surface air of the Northern Hemisphere. Closed red circles: the upper stratosphere (20–40 km height; data from ref. 34); open blue circles: the lower stratosphere (10.1–14.2 km height; data from ref. 16); closed black squares: the surface air (New York; data from ref. 34); brown closed rhombic: the surface air (Beaverton Oregon; data from ref. 34); green closed rhombic: the surface air (Milford Haven; data from ref. 39); purple squares: the surface air (Tsukuba; data from ref. 36,37); brown closed squares: the surface air (Braunschweig; data from ref. 38); green closed circles: the surface air (Prague; data from ref. 7); blue closed rhombic: the surface air (Vilnius; data from ref. 6).

Large data sets on plutonium isotopes in stratospheric and ground-level aerosols is available from the Environmental Measurements Laboratory (EML, USA)^34^, which conducted high-altitude aerosol monitoring programs from the early 1960s to the early 1980s (ref. 35). We used ^238^Pu and ^239,240^Pu activity concentrations in the Northern Hemisphere stratospheric air (20–40 km altitude), in which unreliable results with high measuring uncertainties were removed (Fig. 1). The data obtained by Corcho Alvarado *et al.*^16^ from 1973 to 2009 for the lower stratosphere (10.1–14.2 km altitude) were included in Fig. 1 as well. Further, plutonium isotopes results obtained for surface air at mid-latitude region of the Northern Hemisphere were also included in Fig. 1: New York (USA, 40° 45′N, 74° 00′W), Beaverton Oregon (USA, 45° 32′N, 122° 53′W)^34^, Tsukuba (Japan, 36° 03′N, 140° 08′E)^36^,^37^, Prague (Czech Republic, 50° 04′N, 14° 26′E)^7^, Braunschweig (Germany, 52° 17′N, 10° 33′E)^38^, Vilnius (Lithuania, 54° 42′N, 25° 30′E)^6^ and Milford Haven (UK, 51° 43′N, 5° 02′W)^39^. All plutonium isotope concentrations in surface air were determined at monthly or quarterly basis.

The stratospheric ^239,240^Pu levels showed a maximum in 1963 (Fig. 1) associated with large-scale atmospheric nuclear weapons tests conducted mainly during 1961-62. There were several peaks of the stratospheric ^239,240^Pu in the 1970 s, which correspond to Chinese thermonuclear explosions (total yield of 6.5 Mt in 14 October 1970, 2.5 Mt in 27 June 1973, and 4 Mt in 17 November 1976)^9^. After the 1976 Chinese thermonuclear explosion, the stratospheric ^239,240^Pu concentrations decreased with an apparent stratospheric residence time of 1.3 ± 0.3 y. The apparent residence time of the stratospheric ^239,240^Pu of 2.5–5 y suggested in ref. 16, which is based on the data from 1965 to 2010, is difficult to accept because there are no data available for more than one decade (1987–2003).

A pronounced peak of ^239,240^Pu concentration in surface air of New York occurred at delay of about 17 months after the 1976 Chinese thermonuclear test (Fig. 1). The surface ^239,240^Pu concentration decreased then with the apparent residence time of 1.3 ± 0.3 y (similar to that observed for stratospheric aerosols) until the end of 1980. The surface ^239,240^Pu concentrations showed seasonal variations - a late spring maximum and a winter minimum, in contrast of the stratospheric ^239,240^Pu levels^40^. After the 1980 Chinese nuclear test (total yield of 0.6 Mt in 16 October 1980), a small peak of ^239,240^Pu occurred in the upper stratosphere, which is consistent with the result that most of plutonium from the 1980 Chinese nuclear test was injected into the lower stratosphere and the AME layer just above tropopause^14^. Irrespective of location of surface monitoring sites (New York, Beaverton, Tsukuba, Milford Haven), the surface ^239,240^Pu levels showed marked increase in spring 1981. After 1982, a decrease with the apparent stratospheric residence time of about 1.3 ± 0.3 y was observed until 1984, which means that the surface plutonium until the early 1980s was controlled by the stratospheric inputs. These findings confirm therefore that significant amounts of ^239,240^Pu and fission products in the upper stratospheric air in the 1970s and in the early 1980s were derived from the series of the Chinese nuclear weapons tests.

The ^239,240^Pu levels observed in the lower stratosphere during 2007–2008 were by about two orders of magnitude larger than those observed in surface air^6^,^16^,^31^. The ^239,240^Pu concentrations are expressed per standard cubic meter (15 °C, 101.325 kPa), however, the pressure at 10 km of altitude is about one order of magnitude lower than that in ground-level air. The thermodynamics indicates that sampling volumes in high altitudes are greater than the SCM, therefore it is difficult exactly compare radionuclide concentrations measured at high altitudes with those measured at ground-level air. Another point is a difference of sampling periods between surface and high altitudes measurements. High altitude sampling was usually carried out only for hours, whereas sampling periods of surface air were one to three months. Therefore short-term sporadic events occurring at high altitudes need not be visible in monthly or three months mean values observed at ground-level air.

There are no data available on plutonium concentrations in stratospheric aerosols during the period from June 1986 to October 2004 (Fig. 1). On the other hand, the plutonium isotope levels in surface air were measured in several monitoring sites, most of which were located in Europe. The plutonium concentrations in surface air were as follows: <2.5 to 9.5 nBq m^−3^ during the period from 1986 to 1989 (as quarterly means) at Milford Haven^39^; 0.53 to 8.1 nBq m^−3^ during the period from 1987 to 1998 (as annual means) at Neuherberg^41^; 0.39 to 4.5 nBq m^−3^ during the period from 1990 to 2003 (as quarterly means) at Braunschweig^38^; 0.53 to 217 nBq m^−3^ during the period from 1997 to 2006 (as quarterly means) at Prague^7^; 2.2 to 49 nBq m^−3^ during the period from 2005 to 2006 (as monthly means) at Vilnius (54° 42′N, 25° 30′E)^6^; and 1.7 to 15 nBq m^−3^ during the period from 2001 to 2002 (as monthly means) at Seville (37° 22′N, 5° 59′W)^42^. The results suggest that most of the ^239,240^Pu concentrations in surface air during the period from 1986 to 2006 were in the range of 1 to 10 nBq m^−3^ in the mid-latitude region of the Northern Hemisphere, except of specific events such as a local contamination^7^, Chernobyl accident^6^, and dust events^4^,^5^. The ^239,240^Pu concentrations in surface air did not show decreasing rates, and similar trends were observed for the ^239,240^Pu deposition^4^,^41^. These findings revealed that the ^239,240^Pu concentrations in surface air since 1986 were not under a stratospheric control.

New ^239,240^Pu data for a lower stratosphere are available from October 2004, when they decreased from 0.5 μBq m^−3^ to 0.12 μBq m^−3^ measured in November 2006, in agreement with data obtained during 1986 (ref. 16). Later on, elevated ^239,240^Pu levels were observed in May 2007 (3.9 μBq m^−3^), July 2007 (5.6 μBq m^−3^) and October 2008 (9.7 μBq m^−3^). The last value re-measured in the same day^16^ was, however, only 0.35 μBq m^−3^, indicating a large heterogeneity in the distribution of ^239,240^Pu in the lower stratosphere. Figure 1 clearly shows that the data obtained during 2007–2008 are outside of the ^239,240^Pu stratospheric trend. A new source of plutonium in the stratosphere should be therefore considered.

### Temporal variations of stratospheric ^238^Pu

It has been suggested^16^ that higher ^238^Pu/^239,240^Pu activity ratios observed in stratospheric aerosols in the 2000s may be due to ^238^Pu from burn-up of the US satellite SNAP-9A, which occurred in 1964 at about 50 km altitude over the Southern Hemisphere. The stratospheric behavior of ^238^Pu differed thus from that of ^239,240^Pu because most of the ^238^Pu in the stratosphere after 1964 originated from the burn-up of the SNAP-9A satellite, as the stratospheric inventory of the bomb-derived ^238^Pu was estimated to be only about 2% of its total inventory in 1966^1^,^43^. On the other hand, the stratospheric ^239,240^Pu in the mid 1960s originated mainly from the 1961–62 nuclear weapons testing. In order to trace SNAP-9A satellite-derived ^238^Pu in the stratosphere, it is therefore more appropriate to examine temporal variations of ^238^Pu concentrations in the stratospheric aerosols rather than ^238^Pu/^239,240^Pu activity ratios. The stratospheric ^238^Pu was a good indicator of fine particles injected into the upper stratosphere because ^238^Pu-bearing particles had a physical size distribution with a median at about 0.01 μm of oxide spheres (PuO_2_)^44^.

**Figure 2:**
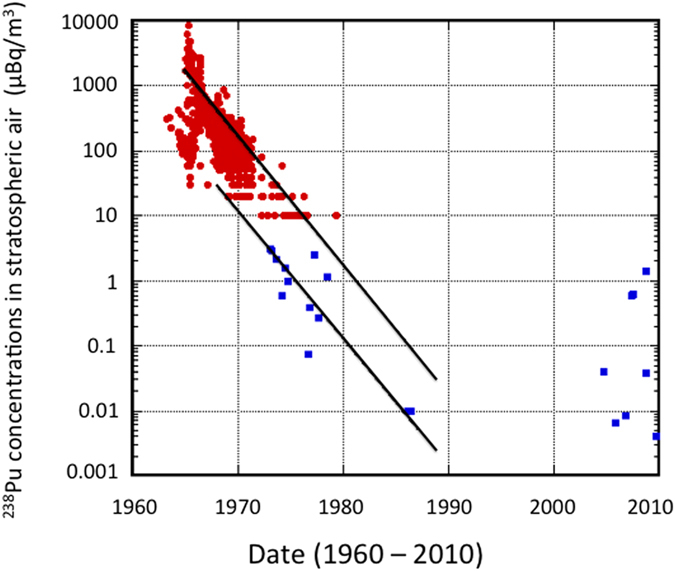
Temporal variations of stratospheric ^238^Pu concentrations in the Northern Hemisphere. Closed circles: the upper stratosphere (20–40 km height; data from ref. 34), closed squares: the lower stratosphere (10.1–14.2 km height; data from ref. 16).

The observed temporal variations of the ^238^Pu concentrations in stratospheric air in the period from 1965 to 1986 showed an exponential decrease (Fig. 2), with an apparent stratospheric residence time of 1.7±0.4 y, consistent with previous results^43^. The result reveals that stratospheric ^238^Pu concentrations showed altitude-depended distribution: higher ^238^Pu concentrations occurred in the upper stratosphere (20–40 km altitude), whereas lower ^238^Pu levels were observed in the lower stratosphere (10.1–14.2 km altitude), which suggests that temporal variations of atmospheric ^238^Pu were controlled by the upper stratospheric ^238^Pu derived from the SNAP-9A burn-up, and partly due to the Chinese nuclear explosions. The ^238^Pu concentrations measured in the stratospheric air in the 2000s were, however, of the same order of magnitude as that during the 1970s and 1980s. It has been suggested^16^ that the high ^238^Pu levels observed during the 2000s were still due to the SNAP-9A burn-up. It is difficult to consider, however, that the supply of the SNAP-9A-derived ^238^Pu from the upper stratosphere in the 2000s was the same level as that during the 1970s, i.e. two orders of magnitude higher than levels observed in 1986, and when between 1986 and 2007 typical global fallout ^238^Pu levels were observed in the lower stratosphere^16^. In fact, most of the SNAP-9A-derived ^238^Pu was deposited on the land surface until the end of 1970s (ref. 14). The decreasing trend in the ^238^Pu levels in the stratosphere up to the mid of the 1980s is also clearly visible in Fig. 2. The elevated ^238^Pu levels observed in the lower stratosphere in the mid of 2000s (ref. 16) should come therefore from other sources than the burn-up of the SNAP-9A satellite.

### Plutonium isotope ratios signatures

The 1961–62 large-scale nuclear tests of the former USSR included a 50 megaton nuclear bomb at Novaya Zemlya^9^, in which a significant amount of nuclear debris was injected into the upper stratosphere. The total explosion yields of the Chinese 1976 and 1980 atmospheric nuclear tests were only 4 and 0.6 megatons^9^, respectively, in which the nuclear debris was injected into the lower stratosphere^14^ only. If the high yield tests in 1961–62 caused higher ^239,240^Pu concentrations in stratospheric aerosols observed in the 2000s, it is expected that stratospheric aerosol plutonium collected in the 2000s would show lower ^238^Pu/^239,240^Pu activity ratios (<0.01) and higher ^240^Pu/^239^Pu atom ratios (>0.3). The ^240^Pu/^239^Pu atom ratios in aerosols observed in the 2010s at 3 km height were, however, near that of soil samples (0.18; ref. 19,26), suggesting thus that a source of plutonium should be a resuspension of global fallout deposited plutonium from the earth surface. Higher ^238^Pu/^239,240^Pu activity ratios and higher ^240^Pu/^239^Pu atom ratios observed in European surface air suggest that source of plutonium should be a resuspension of the Chernobyl-derived plutonium^6^.

The ^241^Pu/^239,240^Pu activity ratio is also a good indicator of plutonium sources in the environment because ^241^Pu has a relatively short half-life (14.4 years). The ^241^Pu/^239,240^Pu activity ratios observed in stratospheric aerosols in the 1970s (12–14) coincided with that observed in deposition samples in 1977 (11; ref. 45). If a major contribution of the stratospheric plutonium was only global fallout, expected ^241^Pu/^239,240^Pu ratios in 1977 and 2005 would be 6 and 2, respectively, assuming that the initial ^241^Pu/^239,240^Pu activity ratios in global fallout was 12.8 (ref. 18). Most of the stratospheric plutonium observed in the 1970s was derived from the Chinese atmospheric nuclear tests, which were conducted in the 1970s. The lower ^241^Pu/^239,240^Pu ratios (ranging from 1.5 to 3.2) observed during the 2000s (ref. 16) in the troposphere (3–13 km altitude), represent therefore aged plutonium derived from global fallout.

The ^239,240^Pu/^137^Cs activity ratios observed in stratospheric aerosols during the 2000s showed a larger variability^16^ than the ratios observed in deposition samples during 2000–2006 (from 0.64 to 8.9 % with a median of 2.5 %; ref. 46). This finding suggests that there are more than two sources of radionuclides in the upper atmosphere, because it is likely that radionuclide composition in the stratospheric aerosols is homogenized throughout a long-time mixing.

## Discussion

### Redistribution of plutonium in the atmosphere

Corcho Alvarado *et al.*^16^ revealed that significant amounts of plutonium isotopes and ^137^Cs existed in the lower stratosphere during the 2000s, which could be transported to the lower troposphere. The recent observations of ^239,240^Pu, ^14^C and ^137^Cs concentrations in surface air^8^,^38^ did not show, however, typical spring maxima, which were observed until the late 1980s when stratosphere-troposphere radionuclide transport was dominant (see also Fig. 1 for ^239,240^Pu). These maxima occurred due to the transport of lower stratospheric air masses containing high radionuclide levels to the upper troposphere. Similar variations have been observed for cosmogenic ^7^Be produced by interactions of cosmic ray particles with nitrogen and oxygen atoms mainly in the lower stratosphere and the upper troposphere. Higher ^137^Cs concentrations in winter months and lower concentrations in summer months are apparent for the last decade, which are more similar to variations of terrigenic ^210^Pb (a decay product of ^222^Rn) than for the ^7^Be variations of the stratospheric origin^8^,^47^,^48^,^49^,^50^. Clearly, there is no more stratospheric influence on the ^137^Cs concentration in surface air, otherwise the ^137^Cs maxima would be observed in the late spring, similarly as the ^7^Be spring maxima, which are still observed in the ground-level air. This change in the ^137^Cs record in the atmosphere is connected with the fact that resuspended ^137^Cs has become important source of tropospheric radioactivity. Similarly ^14^C variations observed in the troposphere after 1990s have been due to ^14^C decreases during winter caused by the Suess effect^8^,^51^. Therefore the absence of spring maxima in ^239,240^Pu, ^137^Cs and ^14^C records during the 2000s suggests that the stratospheric transport does not play anymore a dominant role, but a resuspension from soil or other processes may be responsible for observed radionuclide variations in the atmosphere.

### Global desert dust events

Desert dust events, well known as Saharan dust and Asian dust (Kosa), have been loading large amounts of soil particles into the atmosphere. Saharan dust transport has been known as the biggest global event redistributing aerosols in the atmosphere^52^. The Asian dust clouds, and similarly the Saharan dust clouds were transported in the mid-latitude region around the globe^53^. Since soil particles contain anthropogenic radionuclides primarily derived from global fallout and the Chernobyl accident, Saharan and Asian dusts could cause sporadically increasing anthropogenic radionuclide levels in the atmosphere. Asian dusts were characterized by temporal variations of ^239,240^Pu and ^137^Cs deposition^4^,^46^,^54^,^55^. Similarly, the Saharan dusts cause enhancements of ^137^Cs and ^239,240^Pu concentrations in the atmosphere and in the deposition, which were observed in Monaco^47^,^50^,^56^,^57^,^58^ and south of France^49^. The Saharan dust is characterized by specific isotope ratios (^238^Pu/^239,240^Pu: 0.028, ^241^Pu/^239,240^Pu: 3.1, ^240^Pu/^239^Pu (atom ratio): 0.192, ^241^Am/^239,240^Pu: 0.44, ^239,240^Pu/^137^Cs: 0.027; ref. 49).

It has been shown that during Saharan dust events aerosols can reach 10 km altitude^59^, and thus they can influence radionuclide concentrations in the upper troposphere. Many Saharan dust events were observed by Lidars during the 2000s at altitudes around 10 km (ref. 60, 61, 62). A special year was 2008 when the number of Saharan events exceeded 1000 events/month, with maximum of 2500 events observed in March^63^. The Asian dust clouds generated during the storm in China′s Taklimakan Desert (April 2008) were also lofted to the upper troposphere, to around 8–10 km altitude^60^. Global dust maps ( http://earthobservatory.nasa.gov/GlobalMaps/?eocn=topnav&eoci=globalmaps), which cover events originating not only over Sahara and Asia, but also over North America, Middle East, etc. showed large-scale dust events during 2007 and 2008. The dust outbreaks may thus cause sporadic high ^239,240^Pu levels in the upper troposphere due to high load of surface soil dusts (~mg m^−3^; ref. 49,58) and ^239,240^Pu concentrations in soil (several Bq kg^−1^; ref. 5), and they are therefore possible candidates of observed enhanced plutonium and ^137^Cs levels in the upper and lower troposphere.

### Biomass burning

Other strong candidates of anthropogenic radionuclide variations in the troposphere are forest and grassland fires when huge amounts of particles of submicrometer size are released to the air^64^. The radionuclides derived from the Chernobyl accident as did global fallout had been contaminated in wide areas of Eurasia^9^, which could be lifted back to the atmosphere. The wildfire events could be enhanced by specific meteorological conditions, such as temperature inversions and/or rain events at remote places, causing secondary deposition of ^137^Cs (ref. 65,66). Smoke plumes from biomass fires, could reach several kilometers height, and they can travel distances as long as several thousand of kilometers. They could even penetrate the tropopause, and reach the lower stratosphere^67^,^68^. The biomass burning events could be under specific conditions combined with Saharan dust events causing thus global aerosol impact on the atmosphere. Under specific meteorological conditions they could stay in the atmosphere for several weeks. As around 50 Mha of forest and roughly ten times more grassland are burnt annually, the biomass burning represents important way of radionuclide transport in the environment^69^. The biomass burning plumes originating in Eurasia may redistribute global fallout and Chernobyl deposited sources of anthropogenic radionuclides (e.g. ^137^Cs and ^239,240^Pu) in the atmosphere, which could change concentrations, as well as isotope ratios of these radionuclides in the atmosphere^6^,^31^. An intense biomass burning observed during 2010 in western Russia released around 1 TBq of ^137^Cs to the atmosphere, which enhanced airborne ^137^Cs concentrations up to ~30 μBq m^−3^ at Moscow region, which coincided with aerosol optical measurements^70^.

### Large-scale volcanic eruptions

It has been suggested^16^ that by about three orders of magnitude higher ^239,240^Pu (from 8 to 24 μBq m^−3^) and ^137^Cs (around 1 mBq m^−3^) levels observed in 20 April 2010 in lower troposphere aerosols (altitude 1–3 km) were due to eruption of the Eyjafjallajökull volcano. Sampling carried out the following date showed, however, only global fallout values, as did the sampling carried one year later (30 March 2011) at the altitude of 5.2 and 7.9 km. Elevated radionuclide levels were also reported in 2007 and 2008 in lower stratosphere aerosols (altitude 10.7–12.5 km). On the other hand, no increase in the ^239,240^Pu and ^137^Cs concentrations was observed during 2007–2010 in ground-level aerosols^16^.

A much bigger volcano eruption than the Eyjafjallajökull one was the Mt. Pinatubo (15°08′N, 120°21′E) eruption, which occurred on June 12–16, 1991, and was one of the 20^th^ century′s greatest volcanic eruptions^71^. As a result of this powerful eruption, 15–20 megatons of SO_2_ were injected into the stratosphere, as the eruption columns reached 40 km in altitude. Sulfuric acid and/or sulfate aerosols transformed from SO_2_ can effectively attach radionuclide-bearing particles, and remove them from the stratosphere by a residence time of about 13 months for the Pinatubo aerosol cloud^72^. Unfortunately, there are no stratospheric/tropospheric data available during the 1990s to confirm/discard the volcano hypothesis. As shown in Fig. 1, no enhanced ^239,240^Pu concentration after the Pinatubo eruption, as did ^239,240^Pu deposition^4^,^46^ was observed in surface air. It is likely therefore that remnants of radionuclides derived from the nuclear tests from 1950 to 1980 and from the satellite burn-up in 1964 were already removed from the stratosphere before injection of the Pinatubo aerosol cloud. This has been supported by observations of ^7^Be and ^137^Cs concentrations in summer of 1991 (ref. 47). Therefore there is no obvious evidence of enhanced removal of stratospheric anthropogenic radionuclides due to stratospheric injection of sulfate and ash by large-scale volcanic eruptions.

### Sea-spray effects

It has been pointed out that sea spray may be another potential source of plutonium in the atmosphere^73^,^74^. However, the contribution of plutonium from sea salt to atmospheric plutonium deposition is much lower than that from soil (<0.3%)^4^. It was estimated that a contribution of plutonium from major constituent in sea salt (chloride) in surface air of Tsukuba was below 1 μBq m^−3^. The plutonium/chloride ratio in seawater was 0.5 μBq of Pu per gram of Cl (assuming that plutonium in seawater is homogeneously attached on sea-salt particles)^75^. The calculated maximum contribution of sea-spray plutonium in the atmosphere is less than 0.006 nBq m^−3^, which is by 2–3 orders of magnitude lower than from other potential plutonium sources.

We may conclude that our studies of long-term variations (1964–2010) of plutonium isotopes in the stratosphere and troposphere of the Northern Hemisphere suggest that plutonium levels in ground-level air followed the stratospheric trends until the early 1980s. In the 2000s, plutonium and its isotope ratios in the atmosphere varied dynamically, and sporadic high concentrations of ^239,240^Pu reported for the lower stratospheric and upper tropospheric aerosols may be due to environmental events such as the global dust outbreaks and biomass burning. Long-term measurements of plutonium isotopes in the stratosphere and troposphere revealed that the plutonium concentrations in the stratosphere and the troposphere decreased with apparent residence time of 1.5 ± 0.5 y. The plutonium concentrations in surface air, irrespective of sampling sites in the mid-latitude regions, decreased following the changes in the stratospheric plutonium concentrations.

Anthropogenic radionuclides in the troposphere and the lower stratosphere have been useful tools for better understanding of dynamical processes of aerosols in the atmosphere. Knowledge of their temporal variations has also been important pre-requisites for climate change studies, and assessment of radioecological impacts of nuclear facilities accidents on the atmospheric and terrestrial environments^76^.

## Additional Information

**How to cite this article**: Hirose, K. and Povinec, P. P. Sources of plutonium in the atmosphere and stratosphere-troposphere mixing. *Sci. Rep.* **5**, 15707; doi: 10.1038/srep15707 (2015).
